# Measuring affective reactivity in individuals with autism spectrum personality traits using the visual mismatch negativity event-related brain potential

**DOI:** 10.3389/fnhum.2012.00334

**Published:** 2012-12-19

**Authors:** Leigh C. Gayle, Diana E. Gal, Paul D. Kieffaber

**Affiliations:** Department of Psychology, The College of William and MaryWilliamsburg, VA, USA

**Keywords:** mismatch negativity (MMN), autism spectrum disorders, affect, ERPs, affective disorders

## Abstract

The primary aim of this research was to determine how modulation of the visual mismatch negativity (vMMN) by emotionally laden faces is related to autism spectrum personality traits. Emotionally neutral faces served as the standard stimuli and happy and sad expressions served as vMMN-eliciting deviants. Consistent with prior research, it was anticipated that the amplitude of the vMMN would be increased for emotionally salient stimuli. Extending this finding, it was expected that this emotion-based amplitude sensitivity of the vMMN would be decreased in individuals with higher levels of autism spectrum personality traits as measured by the Adult Autism Spectrum Quotient (AQ). Higher AQ scores were associated with smaller amplitudes of the vMMN in response to happy, but not sad emotional deviants. The fact that higher AQ scores were associated with less sensitivity only to happy emotional expressions is interpreted to be consistent with the negative experience of social interactions reported by individuals who are high on the autism spectrum. This research suggests that the vMMN elicited by deviant emotional expressions may be a useful indicator of affective reactivity and may thus be related to social competency in Autism Spectrum Disorder (ASD).

## Introduction

Autism is a group of pervasive development disorders, often appearing within the first three years of life, that are characterized by atypical development of social and communication skills. Like a growing number of psychological disorders, including schizophrenia, autism is often considered a “spectrum” disorder encompassing a wide variety of symptom profiles and varying degrees of symptom severity. Currently, there are three categories used to group individuals on the autism spectrum. These categories include autistic disorder, Asperger's disorder, and pervasive development disorder not otherwise specified (PDD-NOS), collectively referred to as Autism Spectrum Disorder (ASD) (NIMH, 2011).

Symptoms of ASD include abnormalities in pretend play, social interactions, and verbal and non-verbal communication, as well as patterned or repetitive behaviors and actions such as twirling and banging of the head (Lord et al., 2000). ASD is also typically accompanied by speech and learning difficulties as well as rigid, inflexible routines. These social and communication deficits are most often measured by eye contact, facial expressions and body language, and an evaluation of the child's relationships with peers and family members, (American Psychiatric Association, 2000).

Although the epidemiology of ASD is currently unknown it is commonly linked with neurobiological, neurochemical, and genetic abnormalities (Newschaffer et al., 2007). In the 1950s Dr. Leo Kanner, who originally described autism as a mental disorder, believed that it was a genetically determined phenomenon (Kanner and Eisenberg, 1956). Presently the development of ASD is credited to an interaction between genetic and environmental causes.

Contemporary methods for identifying and diagnosing ASD are rooted in behavioral assessments. These methods typically rely on subjective observations of the child's social and learning behaviors by parents, teachers, and psychiatrists (Lord and Risi, 1998). Standardized tests, such as the Autism Quotient (AQ), the Checklist for Autism in Toddlers (CHAT), Autism Diagnostic Observation Scale (ADOS), and the Autism Diagnostic Interview (ADI), have been developed for the explicit purpose of identifying and quantifying personality and behavioral characteristics thought to occupy the autism spectrum, (Baron-Cohen et al., 2001; NIMH, 2011). Although these behavioral techniques have been used to standardize diagnostic criteria internationally (Lord et al., 1997; Lord and Risi, 1998), their weaknesses include the fact that they ultimately rely on subjective assessments of behavior and that they lack tangible physiological and/or neurological markers that may help to distinguish ASD from other disorders or from socially awkward, but otherwise neurotypical children.

One potentially useful procedure for investigating the integrity of neural mechanisms associated with social or emotional competency is the mismatch negativity (MMN) component of the event-related brain potential (ERP) (Behrmann et al., 2006; Zhao and Li, 2006). The MMN component is typically measured in response to the presentation of a deviant stimulus amidst a sequence of repeated, or “standard,” stimuli. In the auditory domain, the MMN typically occurs 150–200 ms after a deviant stimulus is presented and can last as long as 300 ms (Näätänen et al., 1978; Näätanen, 2007; Garrido et al., 2009). A visual counterpart to the auditory MMN, the visual mismatch negativity (vMMN), is typically observed over parieto-occipital and infero-temporal scalp sites beginning about 140 ms following stimulus onset (Pazo-Alvarez et al., 2003; Maekawa et al., 2005; Czigler et al., 2006; Czigler and Sulykos, 2010).

In both the visual and auditory modalities, the MMN is often considered to be a pre-attentive reaction to change (Dunn et al., 2008), and additionally is thought to be indicative of the comparison of consecutive stimuli, sensory learning, and perceptual acuity (Garrido et al., 2009). Evidence of the pre-attentive nature of the MMN response is typically garnered from findings demonstrating the presence of MMNs in infants (Cheour et al., 2000) and even in comatose patients (Holeckova et al., 2008; Fischer et al., 2010). This quality of the MMN makes it an attractive candidate as an investigative tool for ASD because it can be measured regardless of an individual's level of cognition and/or developmental status, can easily be compared across populations, is independent of language fluency and can even be measured in individuals who are completely non-verbal.

The vMMN has been identified in response to deviances in color, luminance, image contrast, orientation, direction of motion, and spatial frequencies (e.g., Stagg et al., 2004; Näätänen et al., 2007; Li et al., 2012), as well as to more complex visual stimuli such as emotional images or expressions. Variations of the vMMN task have been performed using pictures that elicit emotional responses. In these studies, emotionally neutral images serve as the standard stimulus, and pleasant or unpleasant pictures that have previously been shown to induce either positive or negative emotions are used as deviants. Used in this way, the vMMN is thought to reflect an unconscious, involuntary reaction to change in emotional valence (Kayser et al., 2000; Delplanque et al., 2004, 2005). Zhao and Li (2006) referred to the emotion-elicited vMMN, which is expressed as a larger, or more negative, N170 component and a smaller, or less positive, P250 as the “expressional mismatch negativity” or eMMN (Zhao and Li, 2006; Astikainen and Hietanen, 2009).

Although comparatively little is known about the eMMN, research indicates that it may express hemispheric specialization of emotion processing (Zhao and Li, 2006; Stefanics et al., 2012). However, the nature of this hemispheric specialization is unclear as some findings suggest a right-lateralization in response to positive emotional expressions (Zhao and Li, 2006) and others a left-lateralization for positive emotional expressions (Stefanics et al., 2012). Moreover, recent imaging research further supports the notion that such measures of affective reactivity may be useful as endophenotypic markers of ASD. Spencer et al. (2011) observed significantly reduced activation in brain regions, including the fusiform face area and superior temporal sulcus, in response to happy emotional images in a group of individuals with autism compared with control participants. Most striking was that there was no difference in measures of neural activity between individuals with autism and a group of unaffected siblings of autistic individuals.

The primary aim of the present research was to determine how modulation of the vMMN by emotional expression is related to measures of autism spectrum personality traits in a sample of developmentally typical adults. Using a procedure very similar to the one used by Zhao and Li (2006), vMMN amplitude was measured in response to faces depicting happy or sad emotional expressions amidst a sequence of neutral emotional expressions. One modification to the procedure used by Zhao and Li (2006) was the addition of a non-emotional deviant stimulus, a neutral expression with a green tint added to the image. This non-emotional deviant was used in order to demonstrate that observed variability in the vMMN could be attributed to the emotional content of the deviants. Consistent with prior research (e.g., Zhao and Li, 2006), it was anticipated that the amplitude of the MMN would be increased for emotionally salient stimuli and that the vMMN to emotional expressions in particular would be lateralized in the right hemisphere. Extending prior research, it was expected that this emotion-based amplitude sensitivity would be decreased in individuals with higher levels of autism spectrum personality traits, reflecting a decreased sensitivity to affective expression.

## Methods

### Participants

Forty-five participants (29 Male) without an ASD diagnosis from the College of William and Mary volunteered to participate in this research. The average age of the participants was 19.8 (*SD* = 1.67) years. Each participant provided informed consent and the study was performed in accordance with the rules and regulations of the College of William and Mary's IRB. Eight participants were excluded because of excessive movement artifact in the EEG recordings.

### Measures

After giving informed consent, participants completed the Adult Autism Spectrum Quotient (AQ) while seated behind a privacy screen. The AQ consists of 50 statements regarding social and communication skills, imagination, attention to detail, and sensitivity to change. Participants endorsed each statement with the following ordinal scale: strongly disagree, disagree, agree, and strongly agree. AQ scores were determined in accordance with Baron-Cohen et al. (2001). A score of 25 or above is considered Asperger's and a score of 32 or above meets criteria for a diagnosis of autism. All but one of the participants in this study fell below the level of Asperger's disorder and all of the participants scored below the level of autistic disorder.

### Stimuli

Twelve faces were selected from the NimStim database of standardized expressional faces (Tottenham et al., 2009). The faces included six males and six females, with two black, two white, and two Asian faces within each gender. For each face, one image was selected for each of the neutral, sad, and happy expressions, all with closed mouth expressions.

### Procedure

Participants were seated 37 inches from an LCD monitor inside an electronically shielded Faraday chamber and were fitted with a pair of Eartone 3a insert earphones. Participants were instructed to fixate on a crosshair presented in the center of the monitor and to passively view a series of individual faces while performing an auditory distracter task. For the distracter task, participants were asked to listen to an auditory track of short stories taken from Shel Silverstein's Where the Sidewalk Ends and to count the number of words that began with the letters “T” and “K”. At the end of each block of 115 trials, participants were asked to report the number of words beginning with those letters.

**Figure 1:**
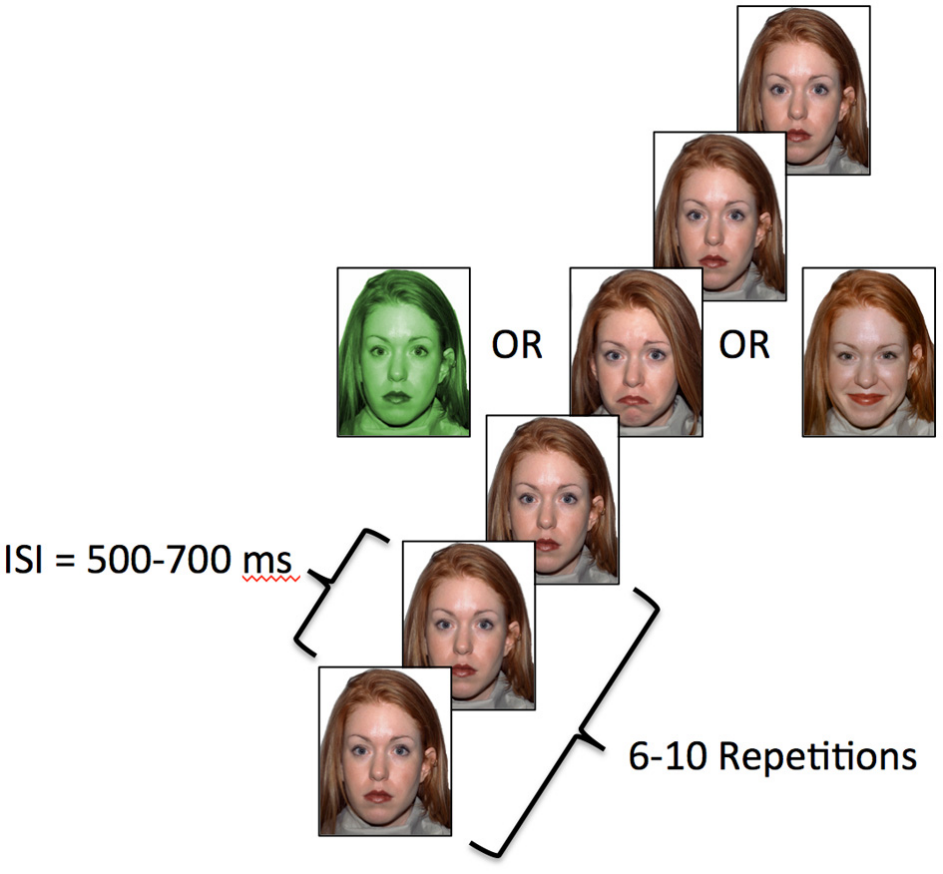
**Task schematic**. Each image was displayed for 150 ms and the ISI was randomized between 500 and 700 ms.

The vMMN procedure included twelve blocks of 115 trials. Each trial consisted of the presentation of 6–10 neutral expressions followed by one deviant expression. Thus, there were 460 instances of each of the three deviant stimulus types over the course of the experiment, which occurred with a probability of ~0.13 on average. The identity of the face was constant within each block of trials, but was counterbalanced across blocks. There were three deviant stimuli presented amidst the sequences of standard stimuli in each trial block (Näätänen et al., 2004). The standard image for each block was a neutral, or non-expressive, face. Two of the deviants were emotional in nature and included faces with happy or sad facial expressions. The third deviant image in each block was the same as the standard image, but with a green tint added (see Figure 1). Occurrence of each of three categories of deviant stimuli was pseudo-randomly ordered and each category was equally represented within a block. Each face remained on screen for 150 ms. The inter-stimulus-interval was randomized to be between 500 and 700 ms and the inter-block interval was 10 s.

### Data acquisition/analysis

Electrophysiological data were recorded continuously at 2000 samples per second using a high-impedance DBPA-1 Sensorium bio-amplifier (Sensorium Inc., Charlotte, VT) with an analog high-pass filter of 0.01 Hz and a low-pass filter of 500 Hz. Recordings were made using a fabric cap bearing 72 Ag-AgCl sintered electrodes. EEG recordings were made using a forehead ground and a reference at the tip of the nose. Vertical and horizontal eye movements were recorded from electrodes placed above and below the eyes and from electrodes placed at the lateral canthi, respectively. All impedances were adjusted to within 0–20 kΩ at the start of the recording session.

EEG data were analyzed off-line using EEGlab. Data were inspected for excessive artifact and channels containing excessive artifacts over a majority of the recording time were interpolated using a spherical spline. Channel interpolation was required for 12 of the 45 participants. Of those 12 participants requiring channel interpolation, two required the interpolation of three channels, one required the interpolation of two channels, and nine required interpolation of just a single channel. Data were then corrected for both horizontal and vertical ocular artifacts using independent component analysis (Jung et al., 2000). Following the removal of ocular artifacts, the data were segmented between −200 and 800 ms with respect to stimulus onset. Following segmentation, data were baseline corrected and filtered using an IIR Butterworth filter with a low-pass frequency cutoff (half-amplitude) of 20 Hz. Individual trials with voltages outside a −100 to 100 μV range were excluded from analysis. Segmented data were then averaged over trials for each of the standard and deviant stimulus presentations.

**Figure 2:**
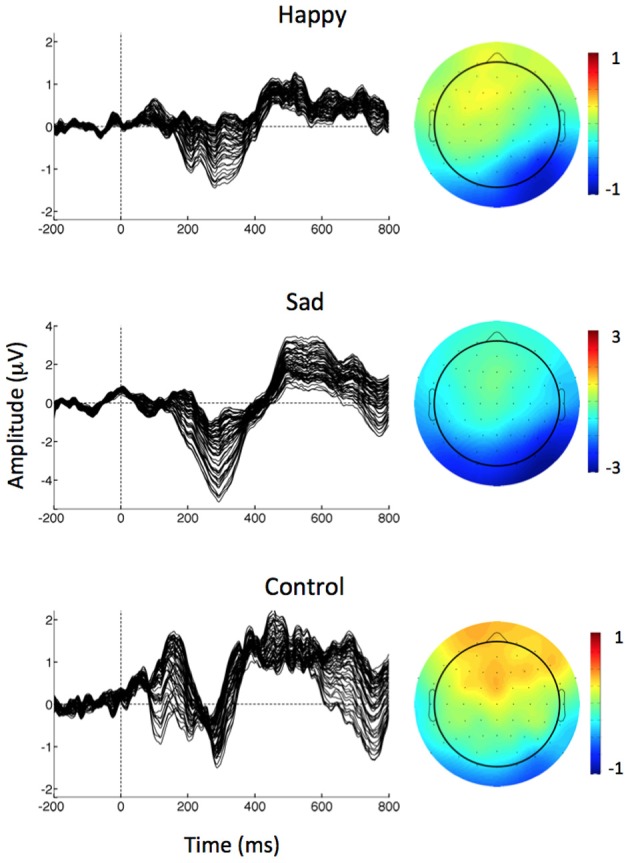
**Left:** Butterfly plot of grand average vMMN waveforms (deviant minus standard) at all electrode sites separately for each of the deviant stimuli. **Right:** Scalp topographies of mean vMMN amplitude (deviant minus standard) over the 150–425 ms epoch for each condition.

vMMN was identified and measured for each condition in the difference waveform generated by subtracting the ERP in response to the Standard image from the ERP in response to the happy, sad, and control deviant images. Combined with prior research (Zhao and Li, 2006), an evaluation of the grand average difference waveforms in Figure 2 informed the decision to measure vMMN as the mean amplitude between 150 and 425 ms at parieto-occipital electrodes (P03, P04, P07, P08). A 3 (Emotion: happy, sad, and control) × 2 [Hemisphere: right (P04, PO8), left (P03, P07)] × 2 [Region: medial (PO3, PO4), lateral (P07, PO8)] repeated measures ANOVA was used to assess amplitude variability across emotional expressions, hemispheres, and medial/lateral regions. Greenhouse–Geisser correction for violations of sphericity was used where appropriate. Guided by the results of the ANOVA, a Pearson correlation coefficient was used to determine the relationship between vMMN amplitude and scores on the AQ.

## Results

### vMMN

**Figure 3:**
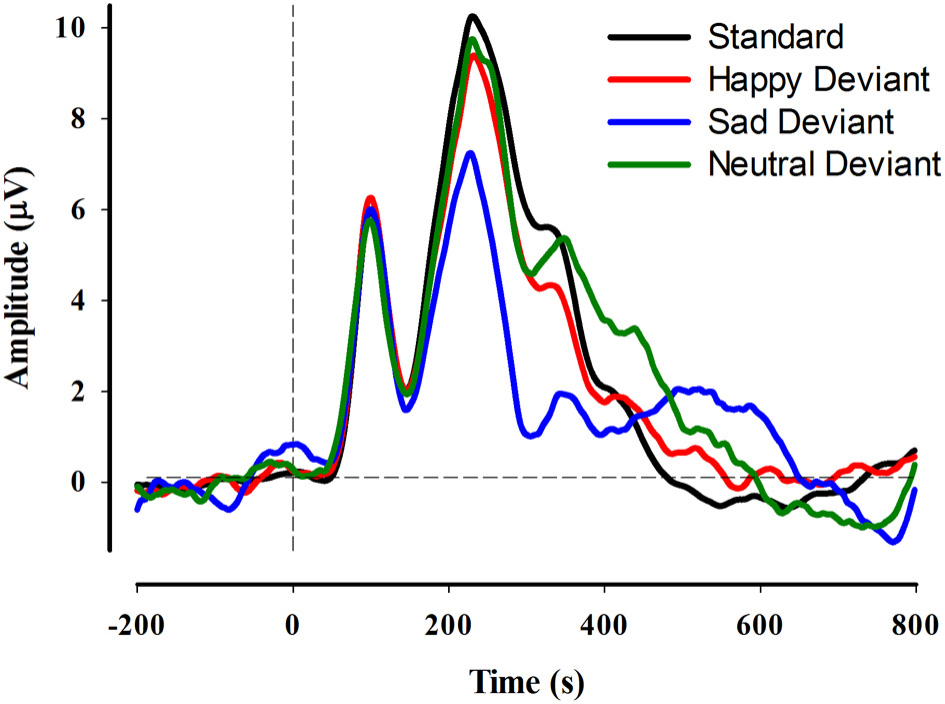
**Grand average ERP waveforms at electrode site PO8 for each of the standard and deviant conditions**.

The grand average ERP in response to standard and deviant stimuli is depicted in Figure 3 for electrode PO8. The repeated measures ANOVA (Emotion × Hemisphere × Region) indicated significant main effects of Hemisphere, Region, and Emotion, each qualified by significant 2-way interactions and a significant 3-way interaction. The main effect of Hemisphere, *F*_(1, 36)_ = 16.4, *p* < 0.001, indicated that vMMN amplitude was larger (more negative) in the right by comparison with the left hemisphere. The main effect of Region, *F*_(1, 36)_ = 8.8, *p* = 0.005, indicated larger vMMN amplitudes in lateral by comparison with medial electrode sites. The main effect of Emotion indicated that vMMN amplitudes were larger in response to sad emotional expressions than either happy (*p* < 0.01) or control (*p* < 0.001) expressions, which were not statistically different from one another.

**Figure 4:**
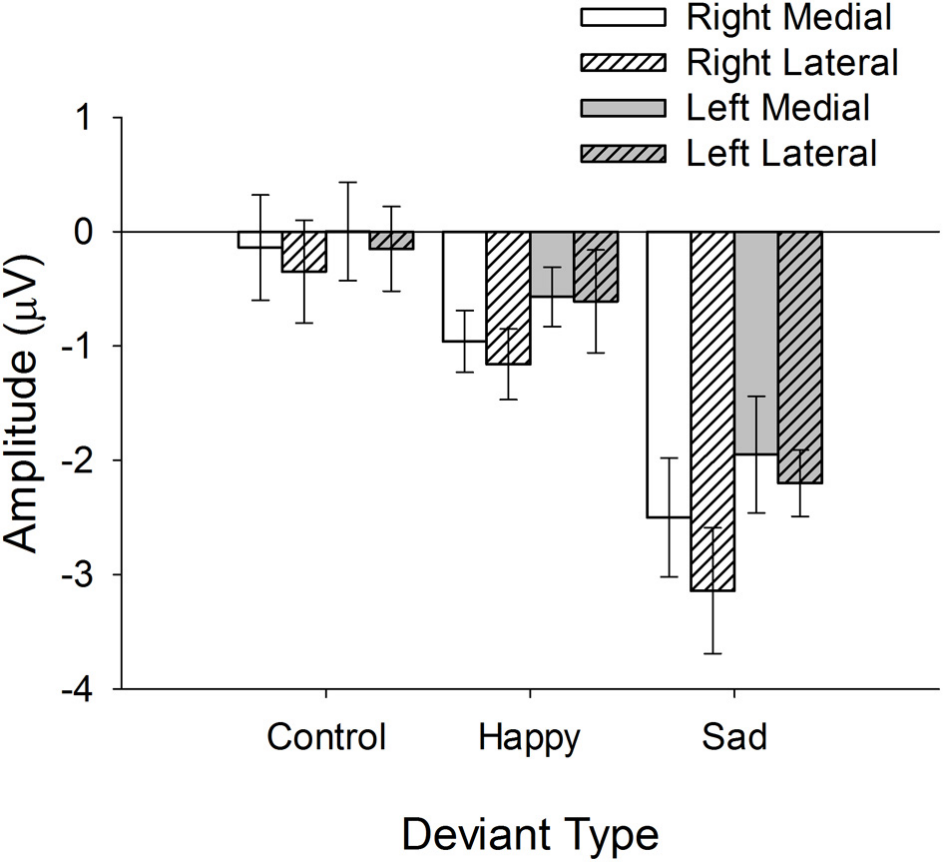
**Mean vMMN (deviant minus standard) amplitudes at each level of Hemisphere, Region, and Deviant Type**.

These main effects were qualified by a number of interactions, including the 3-way interaction between Hemisphere, Region, and Emotion, *F*_(2, 72)_ = 3.3, *p* < 0.05. Figure 4, depicting the mean vMMN amplitudes for each Emotion, Hemisphere, and Region, facilitates the interpretation of this interaction. Inspection of Figure 4 reveals that lateralization of the vMMN to the right hemisphere was increased for sad and happy by comparison with control conditions and that this effect was largest at lateral electrode positions (e.g., PO8).

### Correlation between vMMN amplitude and autism quotient

**Figure 5:**
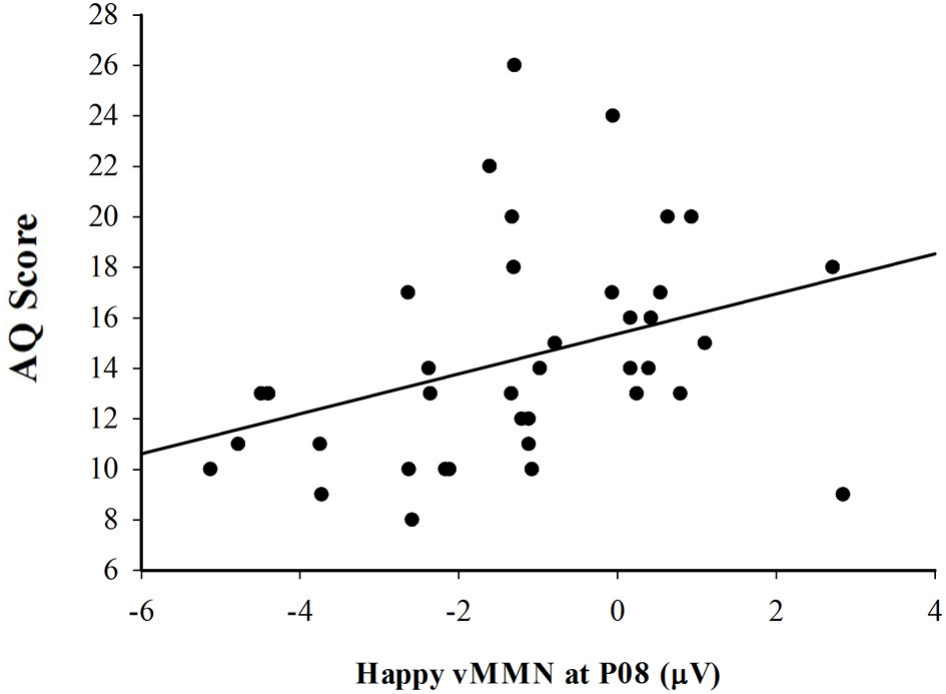
**Scatterplot illustrating the relationship between vMMN (deviant minus standard) amplitude in response to the happy expressions and total AQ score**.

**Figure 6:**
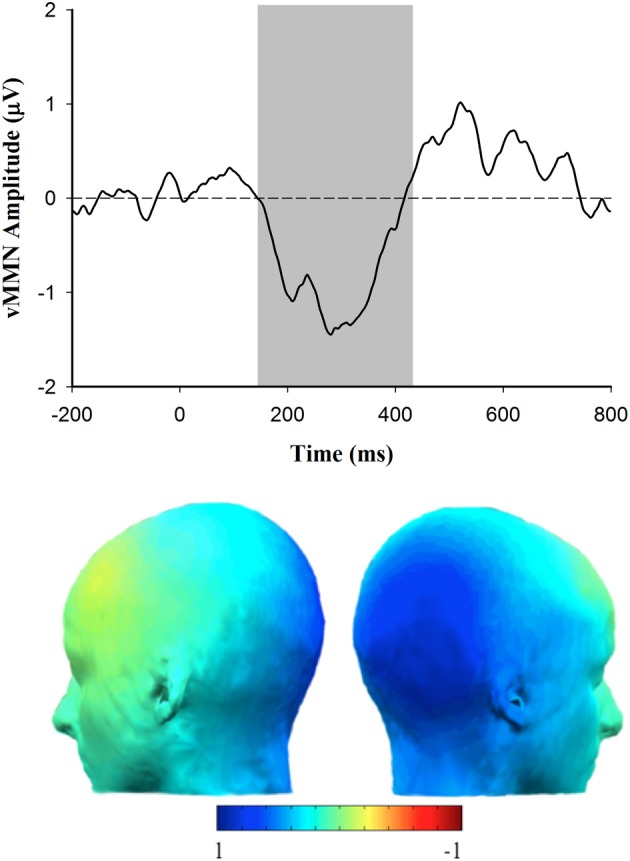
**Grand average vMMN (deviant minus standard) waveform for happy deviants at electrode site PO8 (top) and scalp topography corresponding with the happy vMMN (deviant minus standard) response (shaded area indicates 150–425 ms epoch over which the vMMN difference was quantified)**.

In order to determine how the vMMN amplitude was related to autism spectrum personality traits, a Pearson correlation was used to evaluate the association between AQ score and vMMN amplitude measured over the right lateral (PO8) hemisphere. Consistent with the expectation that vMMN may be useful as an indicator of affective reactivity in ASD, there was a significant positive correlation between the vMMN amplitude to happy deviants and score on the AQ, *r*_(37)_ = 0.343, *p* < 0.05 (see Figure 5). The positive nature of this association indicates that individual's with higher scores on the AQ exhibited smaller (more positive) amplitude vMMN responses to happy emotional expressions. Figure 6 depicts the grand averaged vMMN to happy emotional expressions and the topography of the mean voltage over the 150–425 ms interval used to quantify vMMN amplitude. The correlations between AQ score and vMMN amplitude to sad and control deviants were not statistically significant.

## Discussion

The overarching goal of this research was to determine how modulation of the vMMN by emotional expression is related to autism spectrum personality traits as indicated by the AQ. Electrophysiological data revealed a vMMN to emotional expression that was lateralized to the right hemisphere, a finding consistent with prior research (Blonder et al., 1991; Zhao and Li, 2006; Kimura et al., 2011a,b; Stefanics et al., 2012) and the “right hemisphere hypothesis” (Brood et al., 1998) stating that the right hemisphere is specialized for affective processing. Additionally, a significant positive correlation between vMMN amplitude to happy emotional deviants and the level of autistic personality traits suggests that this measure of affective reactivity may be useful as a tool for measuring affective reactivity in Autism.

Significant differences in vMMN amplitude were also observed between happy and sad emotional expressions, irrespective of the participant's AQ score. Similar effects have been described previously and are thought to be attributable to an inherent “negativity bias,” which describes a predisposition to allocate early processing resources to negative emotional expressions (Stefanics et al., 2012). In fact, Stefanics et al. (2012) report that this negativity bias may appear as early as 195–275 ms following stimulus onset and be localized to the right hemisphere. However, the fact that the correlation between vMMN amplitude to sad expressions and AQ score was not significant, indicates that the impact of “negativity bias” on the vMMN may be independent of the effects of reduced affective reactivity.

The finding that the AQ score was selectively related to vMMN amplitude in response to happy expressions was unexpected in light of related research demonstrating more general deficits of affective processing in ASD, or even a contradictory patterns in some cases (e.g., Blair, 2005; Wallace et al., 2011; Mazefsky et al., 2012; Stefanics et al., 2012). However, this finding is consistent with research indicating low levels of approach motivation and diminished positive affect in individuals diagnosed with ASD (Garon et al., 2009). Additionally, research using startle probe methodology indicates an abnormal profile of affective reactivity in individuals with ASD that is driven by an aberrant psychophysiological response to only positive affect (Wilbarger et al., 2009). Imaging research also indicates that, by comparison with developmentally typical children, individuals with ASD exhibit reduced activation of brain areas like the fusiform face area and superior temporal sulcus in response to positive emotions (Spencer et al., 2011). Remarkably, Spencer et al. (2011) also demonstrate that this reduced affective reactivity is present in unaffected siblings of children with autism compared with controls without a family history of autism. Finally, we interpret a selective reduction of affective sensitivity to positive emotion to be consistent with a negative experience of social interactions in general. Whereas a reduction in sensitivity to negative but not positive affect might actually lead to a more positive overall experience in the context of social interactions, a selective deficit in the processing of positive affect would be expected to lead to an overall negative social experience.

One potential limitation of this study is the fact that the current design, similar to the one used by Zhao and Li (2006), did not counterbalance the designation of standard and deviant stimuli over blocks of the experiment. In other words, the expectancy violation which is thought to be elicited by the appearance of the less-probable emotional or control stimuli and thought to give rise to the vMMN component in the present study is confounded with differences between the happy, sad, and control images on physical dimensions other than emotional valence. This confound complicates direct comparisons between the vMMNs to the various emotional deviants because it is impossible to know whether observed differences are due to the change in affective valence or changes on other physical dimensions of the stimuli. However, these methodological concerns are assuaged by the fact that a similar pattern of results has been shown for happy and fearful emotional expressions using a fully counterbalanced design wherein responses to happy and fearfull emotional expressions each served as “standards” and “deviants” at different points of the experimental procedure (Stefanics et al., 2012), avoiding the problem of confounding physical differences with violations of affective expectancy.

Another important consideration for future research may be gender differences in measures of affective reactivity using the vMMN. This may be important because 64% of the participants in the present sample were male, however, data suggests that males are four times more likely than females to be diagnosed with autism (Lord et al., 2000). The present sample was drawn from the participant pool at a small university, thus, it will also be important to determine that this observed relationship holds in a more diverse population with a more broadly distributed range of traits on the autism spectrum or even an ASD diagnosis.

Notwithstanding these limitations, the present results compel future research to determine how psychophysiological markers, such as the vMMN, may be successfully used to index affective reactivity in individuals with ASD. The fact that the vMMN amplitude was significantly correlated with the measures of behavior indexed by the AQ is important because it complements other recent research indicating that psychophysiological indices like the vMMN are not just epiphenomena, but have explicit behavioral relevance (Stefanics and Czigler, 2012). Moreover, because cognitive-behavioral interventions (CBI) have been shown to be effective in improving social interactions in children with high-functioning autism (Bauminger, 2002), the vMMN may prove to be a useful indicator of treatment efficacy. Finding a tangible neurological marker for ASD could be an important step forward in the development of improved diagnostic procedures and may even reduce the inappropriate labeling of socially awkward, but neurotypical children.

### Conflict of interest statement

The authors declare that the research was conducted in the absence of any commercial or financial relationships that could be construed as a potential conflict of interest.
